# The Δ1-pyrroline-5-carboxylate synthetase family performs diverse physiological functions in stress responses in pear (*Pyrus betulifolia*)

**DOI:** 10.3389/fpls.2022.1066765

**Published:** 2022-11-24

**Authors:** Changqing Ma, Mengqi Wang, Mingrui Zhao, Mengyuan Yu, Xiaodong Zheng, Yike Tian, Zhijuan Sun, Xiaoli Liu, Caihong Wang

**Affiliations:** ^1^ College of Horticulture, Qingdao Agricultural University, Qingdao, China; ^2^ Qingdao Key Laboratory of Genetic Improvement and Breeding in Horticulture Plants, Qingdao, China; ^3^ College of Life Science, Qingdao Agricultural University, Qingdao, China

**Keywords:** P5CS, *Pyrus betulifolia*, transcriptome, stress response, biotic stress

## Abstract

Δ1-Pyrroline-5-carboxylate synthetase (P5CS) acts as the rate-limiting enzyme in the biosynthesis of proline in plants. Although P5CS plays an essential role in plant responses to environmental stresses, its biological functions remain largely unclear in pear (*Pyrus betulifolia*). In the present study, 11 putative pear *P5CSs* (*PbP5CSs*) were identified by comprehensive bioinformatics analysis and classified into five subfamilies. Segmental and tandem duplications contributed to the expansion and evolution of the *PbP5CS* gene family. Various cis-acting elements associated with plant development, hormone responses, and/or stress responses were identified in the promoters of *PbP5CS* genes. To investigate the regulatory roles of *PbP5CS* genes in response to abiotic and biotic stresses, gene expression patterns in publicly available data were explored. The tissue-specific expressional dynamics of *PbP5CS* genes indicate potentially important roles in pear growth and development. Their spatiotemporal expression patterns suggest key functions in multiple environmental stress responses. Transcriptome and real-time quantitative PCR analyses revealed that most *PbP5CS* genes exhibited distinct expression patterns in response to drought, waterlogging, salinity-alkalinity, heat, cold, and infection by *Alternaria alternate* and *Gymnosporangium haraeanum*. The results provide insight into the versatile functions of the *PbP5CS* gene family in stress responses. The findings may assist further exploration of the physiological functions of *PbP5CS* genes for the development and enhancement of stress tolerance in pear and other fruits.

## Introduction

It is well known that abiotic stresses impose severe restrictions on plant growth. These environmental constraints restrict cultivation sites, and diminish agricultural productivity worldwide (Zhu, 2001). To cope with different stresses, plants have evolved multiple mechanisms from physiological, morphological, and molecular perspectives (Tan et al., 2013). Proline plays an essential role in plant growth, development, and stress responses (Anton et al., 2020). It is a compatible solute and a scavenger of reactive oxygen species that provides protection against oxidative damage in plants (Szabados and Savouré, 2010). Free proline is rapidly accumulated in plant cells for adaptation to drought, cold and salinity (Hayat et al., 2012).

Proline biosynthesis involves evolutionarily conserved metabolic pathways in bacteria and higher organisms (Rai and Penna, 2013). Proline biosynthesis uses ornithine or glutamic acid as the substrate, with glutamic acid being preferred under stress conditions (Delauney and Verma, 1993). Δ1-Pyrroline-5-carboxylate synthetase (P5CS) is a key enzyme in the biosynthesis of proline in plants, and it regulates proline content by catalyzing the rate-limiting step in the glutamate pathway (Chen et al., 2013). An increase in P5CS activity can stimulate accumulation of proline to improve osmotic adjustment in plants under environmental stresses (Guan et al., 2014; Anton et al., 2020). P5CS can control proline biosynthesis by transcriptional regulation (Yoshiba et al., 1995). There are two isoforms of P5CS in *Arabidopsis thaliana*, P5CS1 and P5CS2 (Turchetto-Zolet et al., 2009; Funck et al., 2020). Both P5CS isoforms are mainly present in the cytosol, but may be localized in plastids under stress conditions (Székely et al., 2008). However, other P5CS homologues should exist in the plant genome, some of which may be highly stress-induced and essential for proline accumulation.

Since P5CS plays a crucial role in proline biosynthesis, many attempts have been made to enhance proline accumulation by manipulating the *P5CS* gene in order to improve plant stress tolerance. Studies have shown that an increase in P5CS enzyme can induce the accumulation of proline in plants, favoring osmotic adjustment under environmental stresses (Peng et al., 1996; Anton et al., 2020). Over-expression of *P5CS* increased proline content and oxidative stress tolerance in several plants, such as rice (*Oryza sativa*), *A. thaliana*, switchgrass (*Panicum virgatum*), and *Stipa purpurea* under salt and drought stresses (Kumar et al., 2010; Chen et al., 2013; Guan et al., 2020; Yang D. et al., 2021). Moreover, in oriental hybrid lily (*Lilium* spp.), *LhSorP5CS* expression was up-regulated by mannitol and abscisic acid treatments, accompanied by increased proline accumulation (Wang et al., 2017). In addition to abiotic stress, biotic stress also leads to alterations in proline metabolism. For example, *P5CS2* expression was up-regulated in response to infection by *Pseudomonas syringae* in *A. thaliana* (Fabro et al., 2004).

Pear (*Pyrus* spp.) fruits have high nutritional value and are popular among consumers. Pears are reproduced primarily through grafting, with *Pyrus betulifolia* as one of the major rootstocks used in China. Although *P5CS* genes have been identified in *Eugenia uniflora*, *S. purpurea*, rice, and lily (Kumar et al., 2010; Wang et al., 2017; Anton et al., 2020; Yang D. et al., 2021), no comprehensive study of the pear *P5CS* (*PbP5CS*) gene family has been reported. Following the release of the pear genome (Dong et al., 2020), we can now systematically analyze the putative functions of *PbP5CS* genes. In the present study, 11 members of the *PbP5CS* gene family were identified. The *PbP5CS* genes were characterized in terms of gene structures and phylogenetic relationships. Their tissue expression profiles and the expression patterns under different stress conditions were analyzed. The results of *PbP5CS* gene analysis provide insight into the functional role of this gene family in pear.

## Materials and methods

### Identification of *P5CS* genes in the pear genome

Amino acid sequences of the model plant *A. thaliana* P5CS were obtained from the *Arabidopsis* Information Resource database (https://www.arabidopsis.org/). Using these sequences as queries, the pear genome database was screened by BLASTp (E-value <1e^-5^). The complete genome assembly of pear (*Pyrus betulifolia* Bunge.) and the complete proteome sequence file were obtained from the Genome Database for Rosaceae (https://www.rosaceae.org/). Putative *P5CS* genes were confirmed by BLASTp searches of the National Center for Biotechnology Information database (https://www.ncbi.nlm.nih.gov/). Other information obtained from this database included chromosome number, gene accession numbers, predicted masses of proteins encoded by *P5CS* genes, and genomic information. The isoelectric point, grand average of hydropathicity (GRAVY), and molecular weight of P5CS proteins were calculated *via* the ExPasy website (Duvaud et al., 2021). Subcellular locations of pear P5CS proteins were predicted using WoLF PSORT II (Horton et al., 2007).

### Construction of P5CS phylogenetic trees

P5CS amino acid sequences from apple (*Malus domestica*), peach (*Prunus persica*), poplar (*Populus trichocarpa*), and *A. thaliana* were downloaded from Ensembl (https://plants.ensembl.org/index.html). ClustalW v1.83 (Hung et al., 2015) was used for multiple sequence alignments of P5CS proteins. The Muscle module within MEGA 7.0 (Kumar et al., 2016) was used to align the sequences of full-length proteins. Construction of phylogenetic trees based on PbP5CS protein sequences was performed using the neighbor-joining approach with Poisson model, pairwise deletion, and 1000 bootstrap replicates.

### Analysis of conserved motifs, conserved domains, and gene structure

Conserved motifs of all P5CS proteins were identified using the online MEME analysis tool (Bailey et al., 2015) with the maximum number of motifs set at 10, and default values for all other parameters. The NCBI CDD database (https://www.ncbi.nlm.nih.gov/Structure/bwrpsb/bwrpsb.cgi/) was used to analyze the conserved domains of 11 PbP5CS protein sequences, and *P5CS* genes’ domain information data were retained. The information of exon (coding sequence), intron, and untranslated region for 11 *PbP5CS* genes was determined according to the alignments of their sequences in the *P. betulifolia* genome database (https://www.rosaceae.org/species/pyrus_betulifolia/genome_v1.0/). To compare conserved motifs, conserved domains, and gene structures of different groups, TBtools software (Chen et al., 2020) was used for clustering, drawing phylogenetic trees, and mapping conserved motifs of PbP5CS. Exon-intron structures were visualized using Gene Structure Display Server 2.0 (Hu et al., 2014).

### Chromosomal localization and duplication analysis

The chromosomal localization of each *PbP5CS* gene was determined based on physical location information obtained from the pear genome database (https://www.rosaceae.org/species/pyrus_betulifolia/genome_v1.0/). Then, a gene localization and distribution map was drawn using TBtools. Pear genome proteins data were self-compared by BLASTp, and fragment replication type and tandem repeat replication type of *PbP5CSs* were analyzed by MCScanX (Wang et al., 2012). Tandem duplicated genes were identified by analyzing physical locations on specific chromosomes. MCScanX was used to assess syntenic blocks for *PbP5CS* genes, as well as those between pear and *A. thaliana*, between pear and apple, between pear and peach, and between pear and poplar

### Cis-element analysis of *PbP5CS* gene promoters

The promoter sequences of 2000 bp regions upstream of each *PbP5CS* gene-coding region were retrieved from the pear genome database. PlantCARE (Lescot et al., 2002) was then used to annotate elements, and elements related to hormones, stress, growth, and development were selected for location distribution mapping.

### Expression profiles of *PbP5CSs* in various tissues

Expression patterns of *PbP5CS* genes in various tissues were acquired from RNA sequencing (RNA-seq) data available in the NCBI database (https://www.ncbi.nlm.nih.gov/sra/?term=SRP230672/; SRA accession no.: SRP230672). Fragments per kilobase of exon per million mapped reads (FPKM) values were used to estimate gene expression levels. Multi Experiment Viewer (Saeed et al., 2006) was used to evaluate and graphically characterize means of expression values for each gene in all tissues. A heatmap of *PbP5CS* genes was generated using the OmicShare Tool (https://www.omicshare.com/tools/).

### Plant growth conditions and different stress treatments

Pear seeds after vernalization were sown in nutritive soil (65% fertile garden soil, 25% burning soil, 10% fine sand, 0.4% calcium-magnesium-phosphate fertilizer). All materials were kept in a plant incubator. When seedlings grew to the five-leaf stage, they were transplanted into wet vermiculite in pots (7 cm × 7 cm × 10 cm) and kept in an artificial climate room. Pear seedlings received Hoagland solution every 3 days and were grown at 23 ± 2°C with a light intensity of 800 µmol m^-2^·s^-1^. Two-month-old seedlings were used to measure mRNA expression levels of *PbP5CSs* under biotic and abiotic stress conditions. Drought stress of potted pear plants was inflicted by withholding water for 20 days (Yang S. et al., 2021); waterlogging stress was inflicted by submerging plants in water (Yu et al., 2019); salinity-alkalinity stress was performed at a ratio of 1:1.4 NaCl and NaHCO_3_ (Zhang et al., 2020); cold stress was simulated at 4°C (Xi et al., 2011); heat stress was simulated at 40°C (Liu et al., 2013). Pear rust was applied by infecting leaves with *Gymnosporangium haraeanum* Syd. (Li et al., 2006). Pear leaves were taken at 0, 1, 3, and 6 days after abiotic stresses, and at 0, 6, 12, and 24 hours after biotic stress. The samples (with three independent biological replications) were immediately frozen in liquid nitrogen and stored at -80°C until analysis. To explore the gene expression profiles of *PbP5CSs* in response to salt, cold, drought, and *Alternaria alternate* infection, pear RNA-seq datasets were retrieved from published supplemental datasets (SRA accession nos.: SRP077703, SRP287704, SRP148620, and SRP276846).

### Measurements of P5CS enzyme activities

P5CS enzyme activities were measured using a commercial kit (Suzhou Geruisi Biotechnology, Suzhou, China) following the manufacturer’s instructions. Each experiment was independently repeated three times.

### Real-time quantitative PCR analysis

Total RNA extraction from leaf samples was performed using the method of Ma et al. (2022). First-strand cDNA was prepared using PrimeScript RTase (TaKaRa Biotechnology, Dalian, China). Primers for qPCR were designed using Primer Premier 5.0 (Premier Biosoft International, Silicon Valley, CA, USA). Primer sequences are detailed in **Supplementary Table S1**. A LightCycler R 480 SYBR Green Master (Roche, Mannheim, Germany) was used for qPCR assays with a LightCycler R 480 II system (Roche, Rotkreuz, Switzerland). Relative expression levels of the target genes were calculated using the 2^–ΔΔCT^ method (Livak and Schmittgen, 2001) and normalized against the *Actin* gene (GenBank: AB190176).

## Results

### Identification of *P5CS* genes in pear

Based on the conserved domains of protein sequences, 11 P5CS protein sequences were screened and named PbP5CS1−PbP5CS11 according to their chromosomal sequences and positions. Detailed physical and chemical characterizations of PbP5CS proteins are listed in **Table 1**. The 11 PbP5CS proteins have different numbers of amino acids; PbP5CS7 is the shortest (276 amino acids), while PbP5CS4 is the longest (755 amino acids). The molecular weight of PbP5CSs ranged from 31.16 kDa (PbP5CS7) to 82.67 kDa (PbP5CS4). The isoelectric point values of PbP5CS proteins ranged from 6.06 (PbP5CS9) to 8.95 (PbP5CS2). Except for PbP5CS9 and PbP5CS11, the GRAVY values of other PbP5CSs were less than zero. The predicted subcellular localizations were cytoplasm for PbP5CS2, PbP5CS5, and PbP5CS9, endoplasmic reticulum for PbP5CS1 and PbP5CS4, chloroplast for PbP5CS3 and PbP5CS11, and nucleus for the other PbP5CSs.

**Table 1:** General information on *Pyrus betulifolia P5CS* genes.

Gene Name	Gene ID Number^1^	Chr	Start Site	Termination Site	Length(aa)	MW(Da)	PI	GRAVY	Subcellular Localization^2^
*PbP5CS1*	GWHGAAYT001370	1	12601277	12607167	717	77547.02	6.61	-0.052	Endoplasmic reticulum lumen
*PbP5CS2*	GWHGAAYT033428	2	20298570	20301019	338	35870.92	8.95	-0.093	Cytoplasmic
*PbP5CS3*	GWHGAAYT039316	4	20355850	20361403	727	78451.63	6.11	-0.085	Chloroplast
*PbP5CS4*	GWHGAAYT049560	7	21713305	21719297	755	82665.04	6.74	-0.095	Endoplasmic reticulum lumen
*PbP5CS5*	GWHGAAYT011709	12	19348694	19354150	733	79442.93	6.26	-0.051	Cytoplasmic
*PbP5CS6*	GWHGAAYT011728	12	19492870	19502013	450	50284.14	6.72	-0.356	Nuclear
*PbP5CS7*	GWHGAAYT011735	12	19539554	19546307	276	31162.25	6.62	-0.496	Nuclear
*PbP5CS8*	GWHGAAYT011744	12	19602037	19605102	280	31803.65	8.82	-0.329	Nuclear
*PbP5CS9*	GWHGAAYT013594	13	3796252	3799633	338	35712.85	6.06	0.095	Cytoplasmic
*PbP5CS10*	GWHGAAYT017700	14	11096882	11101455	521	56854.74	6.57	-0.493	Nuclear
*PbP5CS11*	GWHGAAYT022995	15	28622901	28626996	335	35632.72	8.83	0.001	Chloroplast

^1^From Pyrus betulifolia Genome Sequence Consortium database. ^2^Predicted using WoLFPSORT (https://www.genscript.com/psort/wolf_psort).

MW, molecular weight; pI, theoretical isoelectric point; GRAVY, Grand Average of Hydropathicity.

### Phylogenetic relationships of PbP5CS members

**Figure 1:**
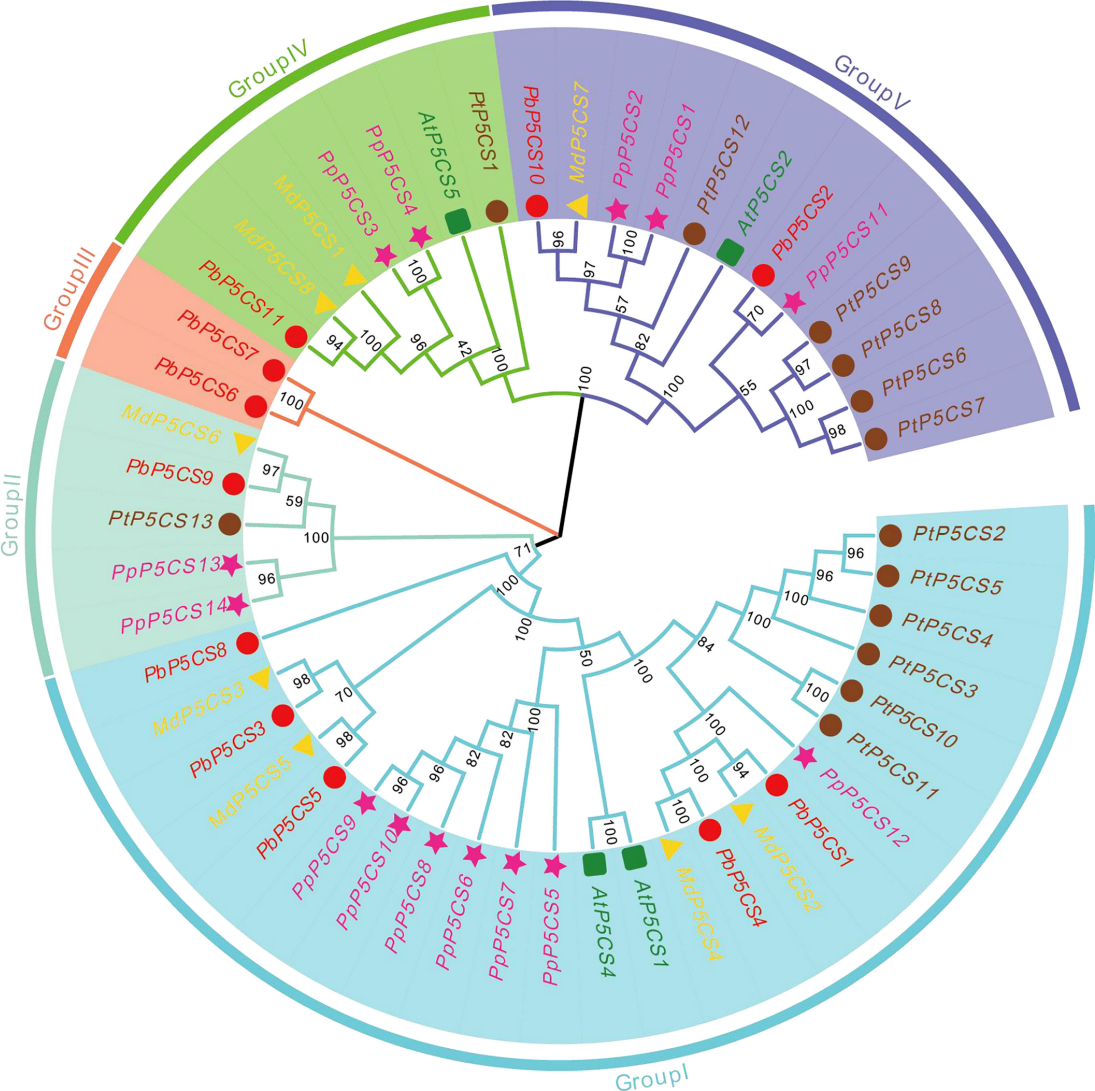
Phylogenetic tree of P5CS proteins constructed using the neighbor-joining method with P5CS domains from pear *Pyrus betulifolia* (red circles), *Malus domestica* (yellow triangles), *Populus trichocarpa* (pink pentagons), *Prunus persica* (brown circles), and *Arabidopsis thaliana* (green squares). Members are divided into Groups I, II, III, IV, and V.

To explore the evolutionary relationships of PbP5CS members, a phylogenetic tree was built using 50 conserved domain sequences of P5CS proteins from pear (11), apple (8), peach (14), poplar (13), and *A. thaliana* (4; **Figure 1**). In the tree, PbP5CS, MdP5CS, PpP5CS, PtP5CS, and AtP5CS were classified into five groups (Groups I, II, III, IV, and V). Notably, *P5CS* genes of woody plants (pear, apple, peach, and poplar) clustered together. Most of the pear P5CSs also clustered together with proteins from *A. thaliana*, consistent with the closer relationship of pear to eudicots. The distribution of pear P5CSs was uneven in these groups. Group I was the largest with 24 members, including nearly half of all pear P5CSs (PbP5CS1, PbP5CS3, PbP5CS4, PbP5CS5, and PbP5CS8). PbP5CS9 and PbP5CS11 were in both Groups II and IV, PbP5CS6 and PbP5CS7 were in Group III, and PbP5CS2 and PbP5CS10 were in Group V.

### Conserved protein motifs and exon-intron structures of *PbP5CS* genes

**Figure 2:**
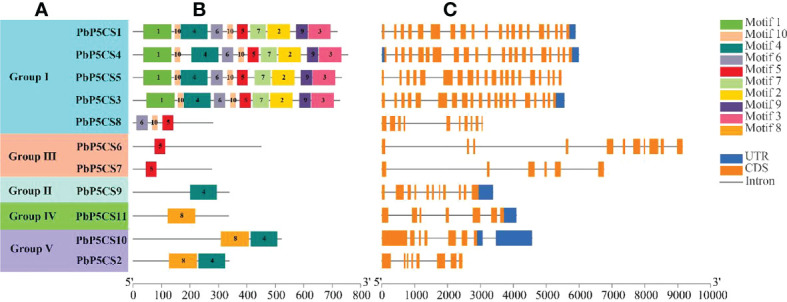
Schematic representation of protein and gene structures of pear *P5CS* (*PbP5Cs*) genes. **(A)** Phylogenetic relationships. **(B)** Motif composition. **(C)** Gene structure. Motifs 1−8 identified using the MEME search tool are marked on protein sequences in each clade (I−V). The length and order of each motif corresponds to the actual length and position in the protein sequences. Coding sequence and untranslated regions are represented by filled orange and dark blue boxes, respectively.

A total of 10 conserved motifs were predicted in PbP5CSs (**Figure 2B**; **Supplementary Table S2**), ranging from 21 to 100 amino acids in length. Interestingly, we observed that Motifs 1, 2, 3, 6, 7, 9, and 10 were present only in Group I members, which might contribute to the functional divergence of *P5CS* genes. Motif 5 was found not only in all members of Group I, but also in PbP5CS6 and PbP5CS7 in Group III, this suggests that PbP5CS6 and PbP5CS7 may have evolved from Group I. Group II only comprised Motif 4, Group IV only contained Motif 8. As Motifs 4 and 8 were found in PbP5CS2 and PbP5CS10 in Group V, PbP5CS2 and PbP5CS10 may have evolved from Groups II and IV (**Figures 2A, B**). We also found that four genes in Group I (*PbP5CS1*, *PbP5CS3*, *PbP5CS4*, and *PbP5CS5*) had more than 20 exons, while all others carried between six and 11 exons (**Figure 2C**).

### Chromosomal locations and homologous genotypes of *PbP5CS* genes

**Figure 3:**
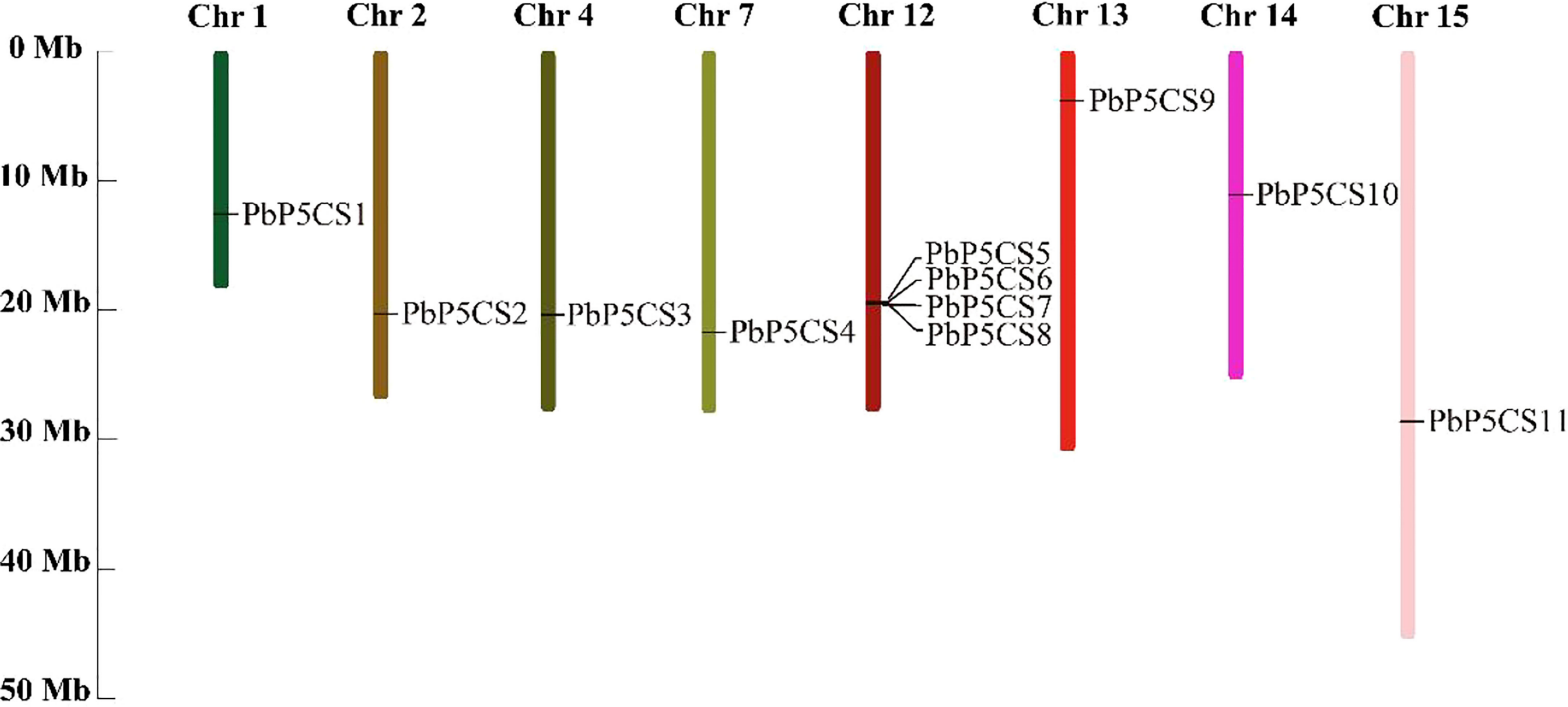
Chromosomal distribution of *PbP5CS* genes. Chromosomal mapping was based on the physical position (Mb) in 17 pear chromosomes. The scale on the left is in megabases (Mb). Chromosome numbers are indicated at the top of each bar.

**Figure 4:**
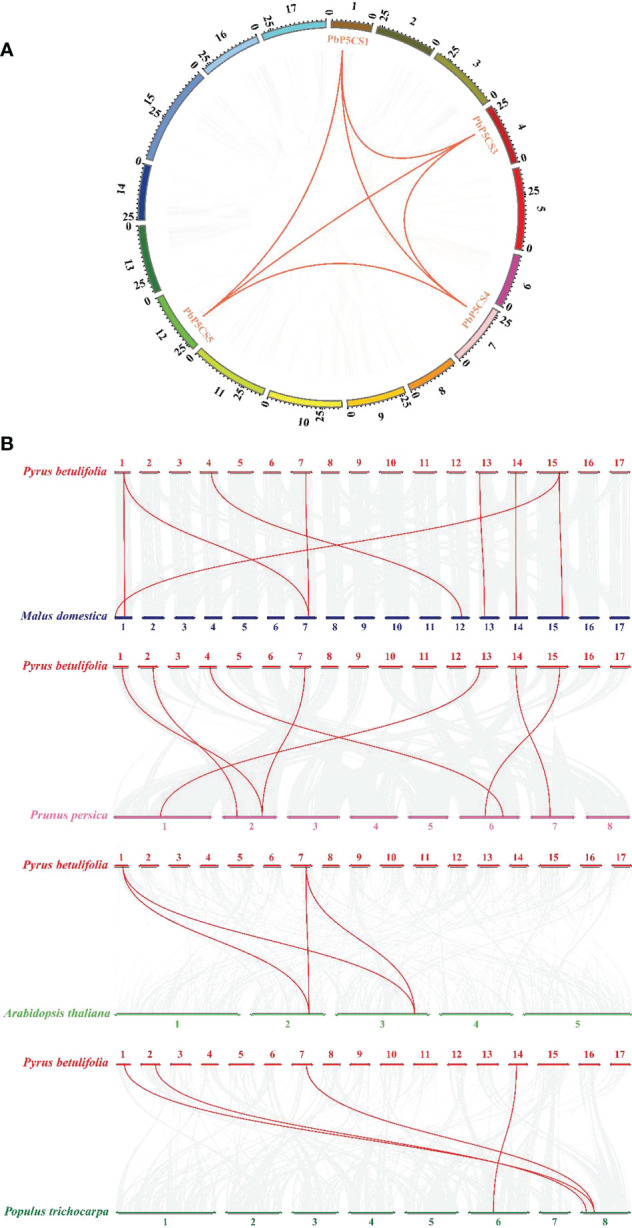
Synteny analysis of *P5CS* genes. **(A)** Synteny analysis of *PbP5CS* genes in pear. Gray lines indicate collinear blocks in the whole *Pyrus betulifolia* genome, and red lines indicate duplicated *PbP5CS* gene pairs. **(B)** Synteny analysis of *P5CS* genes in the *P. betulifolia*, *Populus trichocarpa*, *Arabidopsis thaliana*, *Malus domestica*, and *Prunus persica* genomes. Red lines highlight syntenic *P5CS* gene pairs.

According to their annotated genomic locations, we found that the 11 *PbP5CS*s were widely distributed among the pear chromosomes. Chromosome 12 contained four *PbP5CS* genes, whereas Chromosomes 1, 2, 4, 7, 13, 14, and 15 had only one gene (**Figure 3**). PbP5CS1, PbP5CS3, PbP5CS4, and PbP5CS5 (six pairs) were segmental (**Figure 4A**). In order to further explore the homologous gene relationships of *PbP5CSs*, we compared the physical locations of *P5CS* genes among the genomes of pear, apple, peach, poplar, and *A. thaliana* (**Figure 4B**). *P5CS* genes showed intimate collinear relationships in these five species. *P5CSs* in pear and apple showed the closest collinear relationship, with seven *P5CS* genes in apple sharing a close evolutionary relationship with *PbP5CS* genes. Moreover, six *P5CS* genes in each of peach and poplar, as well as two *P5CS* genes in *A. thaliana*, shared close evolutionary relationships with *PbP5CS* genes (**Supplementary Table S3**).

### Promoter cis-regulatory elements of *PbP5CS* genes

**Figure 5:**
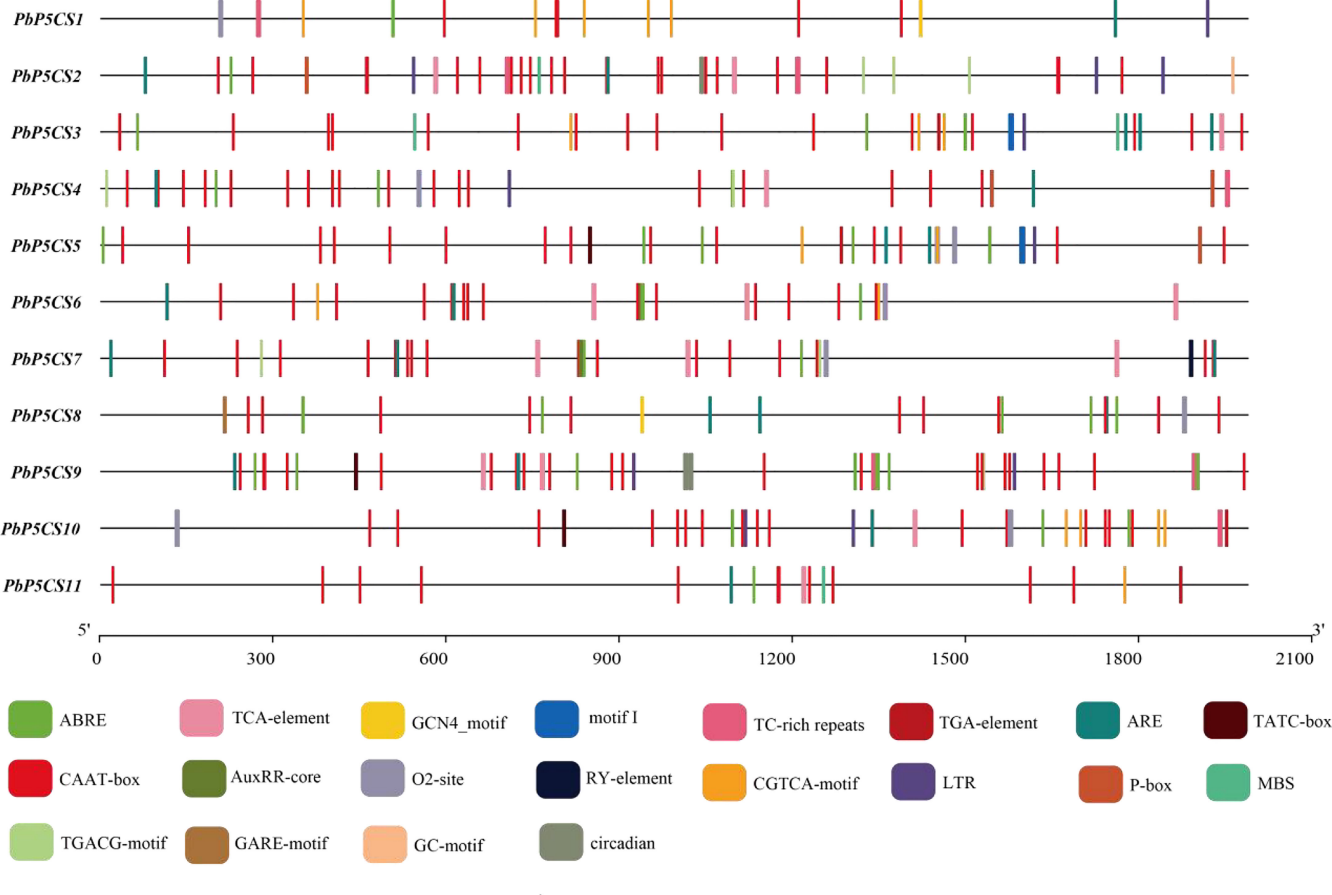
Promoter cis-regulatory element analysis of *PbP5CS* genes. Different colors indicate various promoter elements in *PbP5CS* genes.

To better understand the gene functions and transcriptional regulation of *PbP5CSs*, we analyzed cis-elements in the promoter regions of *PbP5CSs* (**Figure 5**). The conventional promoter element CAAT-box was found in all *PbP5CS* promoters. Various cis-elements related to plant growth, development, and responses to stresses and phytohormones were also identified (**Supplementary Table S4**). Additionally, the conventional promoter element GC-motif was present in *PbP5CS2*, the seed-specific regulation element (RY-element) was found in the promoter of *PbP5CS7*, and the endosperm expression regulation element (GCN4_motif) was observed in the promoters of *PbP5CS1* and *PbP5CS8*. The zein metabolism regulation element (O_2_-site) was identified in the promoters of seven *PbP5CS* genes.

Among the cis-elements that respond to plant hormones, the abscisic acid responsive element (ABRE) was present in the promoter of all *PbP5CS* genes, methyl jasmonate responsive elements (TGACG-motif and CGTCA-motif) were observed in the promoters of all *PbP5CS* genes excluding *PbP5CS8*, and salicylic acid-responsive element (TCA-element) was found in eight *PbP5CS* genes. Gibberellin responsive elements (GARE-motif, P-box, and TATC-box) and auxin responsive elements (AUXRR-core and TGA) were identified in six *PbP5CS* genes. We also found the stress-related cis-acting element (ARE) involved in anaerobic induction in all *PbP5CS* genes. Furthermore, defense and stress response element (TC-rich repeat), drought-inducible response element (MBS), low temperature responsiveness (LTR), and circadian features were identified in the promoters of *PbP5CS* genes (**Figure 5**; **Supplementary Table S4**).

### Tissue-specific expression patterns of *PbP5CS* genes

**Figure 6:**
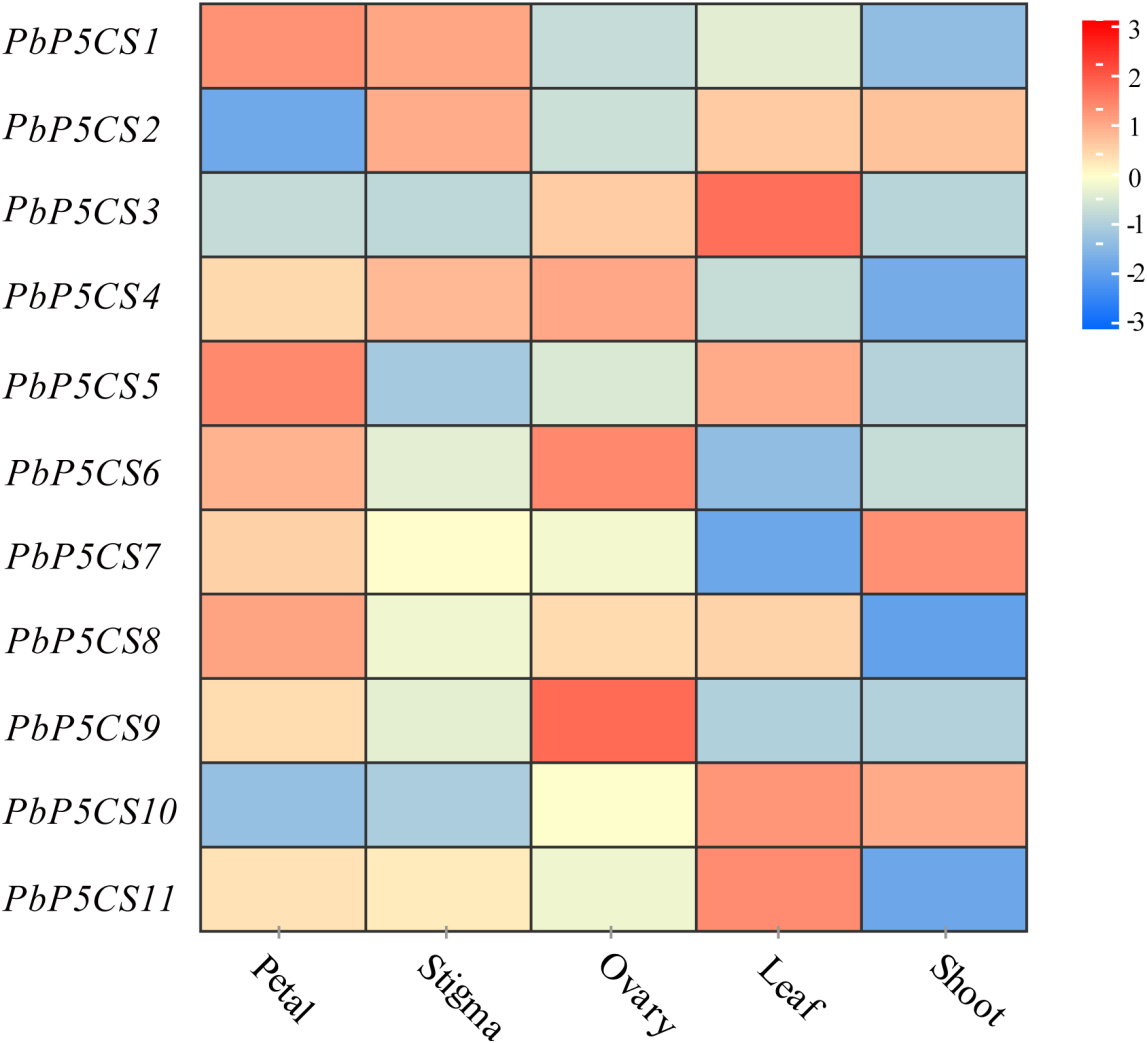
Expression patterns of *PbP5CS* genes in different tissues of pear. Microarray analysis results of *PbP5CS* genes in *Pyrus bretschneideri* ‘Yali’ were downloaded from NCBI GEO DataSets (SRP230672). Red and blue boxes indicate high and low expression levels, respectively, for each gene.

To investigate the putative roles of the *PbP5CS* genes in pear development, we analyzed organic-specific expression patterns of *PbP5CSs*. Expression patterns of *PbP5CS* genes in five different tissues (petal, stigma, leaf, ovary, and shoot) were analyzed using publicly available gene expression data (SRP230672). Some *PbP5CSs* were expressed at considerably high levels in specific tissues (**Figure 6**). For example, three *PbP5CSs* (*PbP5CS1*, *PbP5CS5*, and *PbP5CS8*) displayed higher expression levels in the petal than other tissues, implying that they play important roles in pear petal growth and development.

*PbP5CS1*, *PbP5CS2*, and *PbP5CS4* exhibited high levels of expression in the stigma, which suggests that they may play roles in stigma growth and development. *PbP5CS4*, *PbP5CS6*, and *PbP5CS9* were preferentially expressed in the ovary, which suggests that they may take part in in ovary growth and development. *PbP5CS3*, *PbP5CS5*, *PbP5CS10*, and *PbP5CS11* showed relatively high expression levels in the leaf, which means that they could participate in leaf development. *PbP5CS7* showed higher expression in the shoot than other tissues, which reflects its possible role in shoot growth (**Supplementary Table S5**). These results suggest that *PbP5CS* genes have different expression patterns, and may play diverse roles in pear during growth and development of different tissues.

### Expression patterns of *PbP5CS* genes under abiotic and biotic stresses

**Figure 7:**
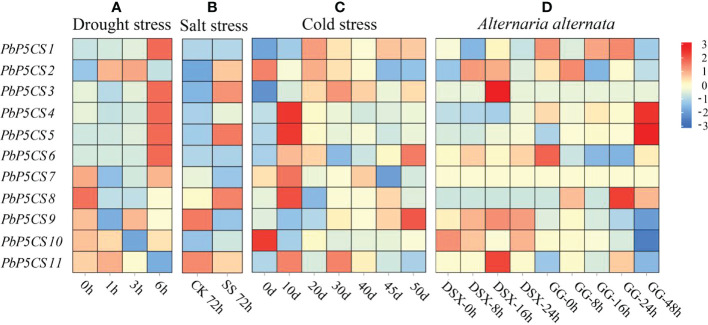
Expression patterns of *PbP5CS* genes in response to abiotic and biotic stresses. **(A)** Gene expression patterns in *Pyrus bretschneideri* at 1, 3, and 6 hours of drought treatment (GEO series SRP148620). **(B)** Gene expression patterns in *Pyrus betulifolia* at 72 hours of salt stress treatment (GEO series SRP077703). CK, control; SS, salt stress treatment. **(C)** Gene expression patterns in *Pyrus bretschneideri* ‘Suli’ at 0, 10, 20, 30, 40, 45, and 50 days of cold treatment (GEO series SRP287704). **(D)** Gene expression patterns in *Pyrus pyrifolia* ‘Deshengxiang’ (DSX) and *Pyrus pyrifolia* ‘Guiguan’ (GG) at 0, 8, 12,16, 24, and 48 hours post-infection by *Alternaria alternate* (GEO series SRP276846). Data are expressed as fragments per kilobase of exon per million mapped reads. Blue and red blocks indicate decreased and increased transcription levels, respectively.

To explore whether the P5CS enzyme plays an essential role under biotic and abiotic stresses, we measured the enzyme activities of P5CS under drought, waterlogging, salinity-alkalinity, cold, heat, and *G. haraeanum* infection (**Supplementary Figure S1**). The enzyme activities of P5CS gradually increased under the different stresses. We then investigated expression patterns of *PbP5CS* genes in response to different stresses. With regard to abiotic stresses, RNA-seq datasets for pear subjected to drought (SRP148620), salt (SRP077703), and cold (SRP287704) were explored. In general, expression levels of *PbP5CS1*, *PbP5CS3*, *PbP5CS4*, *PbP5CS5*, *PbP5CS6*, and *PbP5CS11* were up-regulated by all abiotic stress treatments, indicating potential roles of these *PbP5CSs* in abiotic stress responses (**Figure 7**; **Supplementary Table S6**). Under drought treatment, *PbP5CS1*, *PbP5CS2, PbP5CS3*, *PbP5CS4*, *PbP5CS5*, *PbP5CS6*, *PbP5CS7*, *PbP5CS9*, and *PbP5CS11* were up-regulated, but *PbP5CS8* and *PbP5CS10* were repressed in response to short-term drought stress (**Figure 7A**). Furthermore, *PbP5CS2*, *PbP5CS3*, *PbP5CS5*, and *PbP5CS8* were significantly up-regulated at 72 hours of NaCl treatment (**Figure 7B**). *PbP5CS6* and *PbP5CS9* were significantly induced at 50 days of cold treatment (**Figure 7C**). These results indicate that the responsive *PbP5CSs* may be involved in plant defense mechanisms under both short- and long-term abiotic stress.

To explore the potential roles of *PbP5CS* genes in responses to biotic stresses, we investigated RNA-seq datasets from the infection experiment of pear with *A. alternate* (SRP276846). *PbP5CS1* and *PbP5CS2* were significantly up-regulated at 24 and 8 hours post-infection, respectively. *PbP5CS3*, *PbP5CS9*, and *PbP5CS11* showed significant up-regulation at 16 hours post-infection. *PbP5CS4*, *PbP5CS5*, and *PbP5CS8* were markedly induced at 48 hours post-infection. However, *PbP5CS6* and *PbP5CS10* were distinctively repressed following *A. alternate* infection, whereas *PbP5CS7* did not respond to infection (**Figure 7D**).

**Figure 8:**
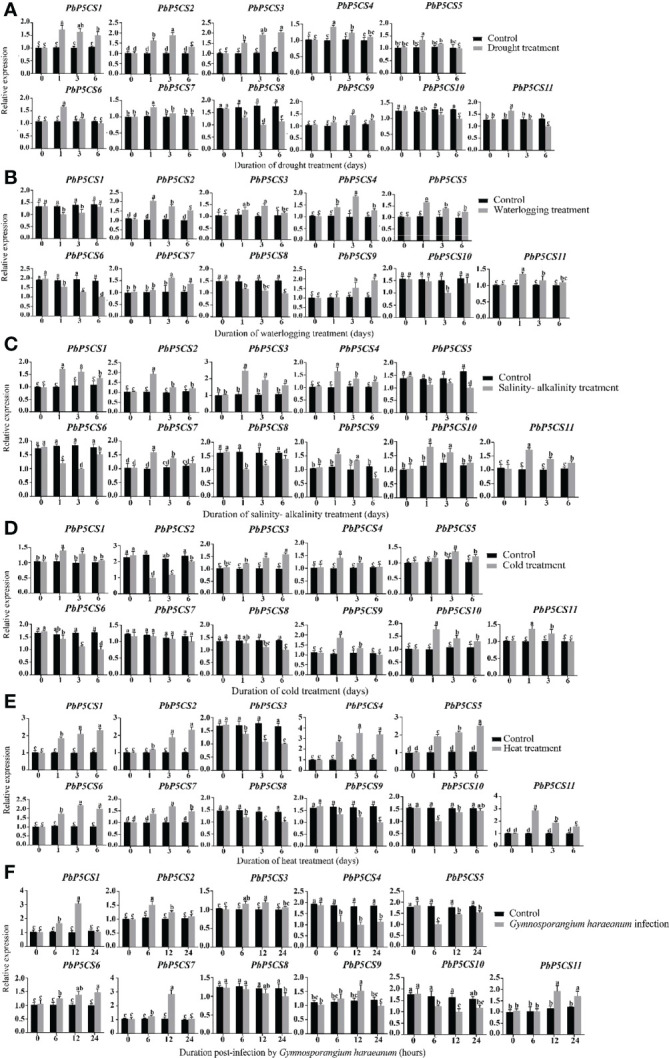
Real-time quantitative PCR (qPCR) analysis of pear *P5CS* genes in response to **(A)** drought, **(B)** waterlogging, **(C)** salinity-alkalinity, **(D)** cold, **(E)** heat, and **(F)** *Gymnosporangium haraeanum* infection stresses. Data were normalized against expression levels of the *Actin* gene. Mean values were calculated from three independent replicates. Vertical bars indicate standard error of the mean. Different lowercase letters indicate significant differences between treatments according to Fisher’s least significant difference (P < 0.05).

To validate previous RNA-seq data and reveal more details of *PbP5CSs* in stress responses, we investigated the transcription levels of *PbP5CS* genes under different environmental stresses, including drought, waterlogging, salinity-alkalinity, cold, heat, and *G. haraeanum* infection. Under drought stress, nine (*PbP5CS1, PbP5CS2, PbP5CS3, PbP5CS4, PbP5CS5, PbP5CS6, PbP5CS7, PbP5CS9*, and *PbP5CS11*) and two (*PbP5CS8* and *PbP5CS10*) *PbP5CS*s were up-regulated and down-regulated, respectively (**Figure 8A**). Moreover, seven (*PbP5CS2, PbP5CS3, PbP5CS4, PbP5CS5, PbP5CS7, PbP5CS9*, and *PbP5CS11*) and four (*PbP5CS3, PbP5CS8, PbP5CS9*, and *PbP5CS10*) *PbP5CS* genes were respectively up-regulated and down-regulated under waterlogging treatment (**Figure 8B**). Similarly, eight *PbP5CS* genes (*PbP5CS1, PbP5CS2, PbP5CS3, PbP5CS4, PbP5CS7, PbP5CS9, PbP5CS10*, and *PbP5CS11*) showed increased expression levels and three *PbP5CSs* (*PbP5CS5, PbP5CS6*, and *PbP5CS8*) were down-regulated by salinity-alkalinity treatment (**Figure 8C**). Additionally, the transcription levels of seven *PbP5CS* genes (*PbP5CS1, PbP5CS3, PbP5CS4, PbP5CS5, PbP5CS9, PbP5CS10*, and *PbP5CS11*) were increased under cold stress (**Figure 8D**). Under heat stress, seven *PbP5CS* genes (*PbP5CS1, PbP5CS2, PbP5CS4, PbP5CS5, PbP5CS6, PbP5CS7*, and *PbP5CS11*) were up-regulated, and the remaining four *PbP5CSs* (*PbP5CS3, PbP5CS8, PbP5CS9*, and *PbP5CS10*) were down-regulated (**Figure 8E**).

Pear rust caused by *G. haraeanum* is one of the main diseases affecting pear production. To understand the potential functions of *PbP5CS* genes in response to biotic stress, transcript levels of 11 *PbP5CSs* were measured by qPCR in pear subjected to *G. haraeanum* infection. Seven (*PbP5CS1, PbP5CS2, PbP5CS3, PbP5CS6, PbP5CS7, PbP5CS9*, and *PbP5CS11*) and four (*PbP5CS4, PbP5CS5, PbP5CS8*, and *PbP5CS10*) *PbP5CS* genes were up-regulated and down-regulated, respectively (**Figure 8F**). Interestingly, *PbP5CS4* and *PbP5CS11* were also strongly induced by drought, waterlogging, salinity-alkalinity, cold, and heat, suggesting that these two genes might be candidate genes for mitigating abiotic stresses. In particular, *PbP5CS11* was induced dramatically by various abiotic and biotic stresses (**Figure 8**).

## Discussion

*P5CS* genes play key roles in biochemical and physiological processes in response to various stressors in plants (Chen et al., 2013; Yang D. et al., 2021). Therefore, studying the functions of *P5CS* gene families in inhospitable environments can provide valuable information on the mechanisms underlying plant adaptation. In the current work, we performed a genome-wide analysis of *P5CSs* in pear by considering their gene structures, phylogenetic relationships, cis-acting elements, linkage group organization, and duplication events. We also explored their possible roles in plant tissues and responses to stress. The genome-wide results for *PbP5CS* genes not only provides novel insights into their physiological functions, but also a foundation for functional research on these genes during pear growth and development.

The expansion of gene family members is driven by gene duplication events, either segmental or tandem duplications, during plant evolution (Guo et al., 2014; Wang et al., 2022). Most plants have two *P5CS* isoforms, as demonstrated for *Phaseolus vulgaris*, *Lotus japonicus*, and *Brassica napus* (Xue et al., 2009; Chen et al., 2013; Signorelli and Monza, 2017). In the present work, 11 *P5CS* genes were identified in the pear genome (**Table 1**; **Figure 1**), more than apple (8) and *A. thaliana* (4), but fewer than poplar (13) and peach (14). The variation might be due to gene duplication differences, considered a fundamental driving force in the evolution of genomes (Kong et al., 2007). Gene duplications can provide raw materials for new genes, leading to the emergence of new functions.

Our phylogenetic analysis of P5CS proteins among pear, apple, peach, poplar, and *A. thaliana* showed that the proteins formed species-specific clusters (**Figure 1**). This result indicates that P5CS proteins have been highly conserved during evolution. Furthermore, there were six segmental duplication pairs (*PbP5CS1*/*PbP5CS3*, *PbP5CS1*/*PbP5CS4*, *PbP5CS1*/*PbP5CS5*, *PbP5CS3*/*PbP5CS4*, *PbP5CS3*/*PbP5CS5*, and *PbP5CS4*/*PbP5CS5*) in pear (**Figure 4A**). These results demonstrate that segmental duplication plays a vital role in driving the expansion of the pear *PbP5CS* gene family. The synteny analysis of *P5CS* genes in pear, apple, peach, poplar, and *A. thaliana* showed that *PbP5CS* genes shared higher homology with *P5CS* genes in apple than in peach, poplar, and *A. thaliana* (**Figure 4B**; **Supplementary Table S3**). However, *PbP5CS1* and *PbP5CS4* were collinear with the *P5CS* genes of the other four species (**Supplementary Table S3**), indicating that *PbP5CS1* and *PbP5CS4* in different plants may have evolved from a common ancestor.

Variation in introns and exons plays is essential for the evolution of different genes (Mustafin and Khusnutdinova, 2015; Rogers, 1990). Introns play major roles in gene evolution (Rose, 2008). Our analysis of *P5CS* gene structure revealed that all *PbP5CS* genes contained different numbers of exons and introns (**Figure 2C**), indicating functional diversity among *PbP5CS* genes. In addition, cis-regulatory element analysis revealed the presence of a series of abiotic/biotic stress responsive cis-acting elements, such as ARE, ABRE, MBS, LTR, and AuxRR-core, in the promoter regions of *PbP5CS* genes (**Figure 5**; **Supplementary Table S4**). This implies that the *PbP5CS* genes perform potential functions in response to abiotic and biotic stresses. The different cis-regulatory elements in *P5CS* genes presumably allows them to exert diverse effects on plant growth and development, including under different stress conditions.

Proline has been shown to be critically involved in a number of plant developmental processes, such as pollen fertility, root elongation, embryo development, and floral transition (Trovato et al., 2018). P5CS1 predominantly contributes to stress-induced proline accumulation, and P5CS2 is mainly involved in plant growth and development (Funck et al., 2020). For example, an increase in proline content was accompanied by markedly high expression of *BnP5CS* in flowers of *B. napus*, suggesting possible contribution of proline to flower development (Xue et al., 2009). In pear, eight *PbP5CS* genes (*PbP5CS1*, *PbP5CS4*, *PbP5CS5*, *PbP5CS6*, *PbP5CS7*, *PbP5CS8*, *PbP5CS9*, and *PbP5CS11*) displayed high expression levels in the petal (**Figure 6**), which further indicates that proline is a key factor in floral development (Trovato et al., 2018). Furthermore, *PbP5CS1*, *PbP5CS2*, and *PbP5CS4* were highly expressed in the stigma, *PbP5CS4*, *PbP5CS6*, and *PbP5CS9* showed relatively high expression levels in the ovary, and *PbP5CS3*, *PbP5CS10*, and *PbP5CS11* were predominantly expressed in the leaf (**Figure 6**). All these *PbP5CS* members likely function in the particular pear tissues.

Proline accumulation is mainly regulated by the P5CS enzyme in plant cells under stress conditions (Yang D. et al., 2021). Increasing evidence demonstrates that *P5CS* genes participate in plant development, biological regulation, and stress responses, and they play an essential role in plant resistance to different abiotic stresses (Zhang et al., 1995; Parida et al., 2008; Bandurska et al., 2017). P5CS is a key enzyme enhancing oxidative stress tolerance in plants under salt and drought stresses (Kumar et al., 2010; Rai and Penna, 2013). P5CS activity and expression levels were up-regulated in barley (*Hordeum vulgare*), cotton (*Gossypium hirsutum*), and *S. purpurea* under drought stress conditions (Parida et al., 2008; Bandurska et al., 2017; Yang D. et al., 2021). In pear, P5CS enzyme activity was induced under six different stresses (**Supplementary Figure S1**). The expression of *PbP5CS1, PbP5CS2, PbP5CS3, PbP5CS4, PbP5CS5, PbP5CS6*, and *PbP5CS11* was significantly induced in response to drought stress according to RNA-seq and qPCR data (**Figures 7**, **8**), indicating roles for these genes in drought stress tolerance by regulating P5CS enzyme activities. Moreover, most pear P5CSs (PbP5CS1, PbP5CS2, PbP5CS3, PbP5CS4, PbP5CS5, PbP5CS6, PbP5CS8, PbP5CS10, and PbP5CS11) clustered together with proteins from *A. thaliana* (**Figure 1**). The *AtP5CS* gene was induced by high salt treatment in *A. thaliana* (Yoshiba et al., 1995). Similarly, *PbP5CS2*, *PbP5CS4*, and *PbP5CS10* expression was induced in response to salt and salinity-alkalinity stresses, according to RNA-seq and qPCR data (**Figures 7**, **8C**), indicating putative functions for these genes in pear salt or salinity-alkalinity stress tolerance. Furthermore, *AmP5CS* was rapidly initiated by heat stress in grey mangrove (*Avicennia marina*) (Liu and Wang, 2020). *PvP5CS* was prominently up-regulated in common bean (*Phaseolus vulgaris*), which enhanced tolerance under cold stress (Atienza et al., 2004; Chen et al., 2009; Chen et al., 2013). *CpP5CS* can be induced by both heat and cold stress in papaya (*Carica papaya*) (Zhu et al., 2012). In the current work, P5CS enzyme activity was gradually increased, and the expression of *PbP5CS1*, *PbP5CS4*, *PbP5CS5*, and *PbP5CS11* was significantly induced by heat and cold. Additionally, the enzyme activity of P5CS was induced, and *PbP5CS2*, *PbP5CS3*, *PbP5CS4*, *PbP5CS5*, *PbP5CS7*, *PbP5CS9*, and *PbP5CS11* were up-regulated by waterlogging stress (**Supplementary Figure S1**; **Figure 8**). The collective results indicate that these *PbP5CS* genes also regulate P5CS enzyme activities to mitigate various abiotic stresses.

Regarding biotic stresses, *AtP5CS2* participates in the *A. thaliana* hypersensitive response induced by avirulent *Pseudomonas* spp. (Fabro et al., 2004). Herein, *PbP5CS1*, *PbP5CS2*, *PbP5CS3*, *PbP5CS6*, *PbP5CS7*, *PbP5CS9*, and *PbP5CS11* were up-regulated in response to *G. haraeanum* infection (**Figure 8**). Accordingly, these *PbP5CSs* might participate in the pathogen response pathway. However, *PbP5CS4*, *PbP5CS5*, *PbP5CS8*, and *PbP5CS10* were down-regulated in response to *G. haraeanum* infection, suggesting that the four genes may function through different mechanisms to protect against biotic stimuli. All identified *PbP5CS* members were differentially regulated by both biotic and abiotic stresses, indicating that these genes are likely to mediate plant defense mechanisms in pear. Currently, the biological functions of most *PbP5CS* genes in plant developmental and defense processes remain unknown. The present bioinformatic and expression analyses of *PbP5CS* genes provide valuable information for screening candidate genes, and the results are helpful to further investigate the functions of this gene family in pear.

## Conclusions

Eleven *PbP5CS* genes were identified in pear, and a systematic study of the *PbP5CS* gene family was carried out. The comprehensive analyses encompassed conserved domains, gene structures, and phylogenetic relationships, in addition to gene duplications, chromosome locations, cis-acting elements, and expression patterns. There were various cis-acting elements in the *PbP5CS* promoter sequences, suggesting that *PbP5CSs* act in complex networks regulating plant development and responses to stresses. Transcriptome and qPCR analyses revealed that *PbP5CS* genes are likely to take part in plant response to biotic and abiotic stresses. Our genome-wide analysis of *PbP5CSs* provides evidence for the functions of this gene family in pear. Further studies on *PbP5CS* genes are underway to verify their functions in stressed environments.

## Data availability statement

The datasets presented in this study can be found in online repositories. The names of the repository/repositories and accession number(s) can be found in the article/**Supplementary Material**.

## Author contributions

CM and CW conceived and designed the research. MW, MZ, MY, XZ, YT, ZS, and XL performed the experiments, conducted the field work, and analyzed the data. MC and MW wrote the manuscript. All authors contributed to the article and approved the submitted version.

## Funding

This study was supported by the Funds for Modern Agricultural Industry Technology System in Shandong Province (SDAIT-06-06), the Shandong Provincial Natural Science Foundation (ZR2019BC038), and the High-level Scientific Research Foundation of Qingdao Agricultural University (Grant 663/1121043), China.

## Conflict of interest

The authors declare that the research was conducted in the absence of any commercial or financial relationships that could be construed as a potential conflict of interest.

## Publisher’s note

All claims expressed in this article are solely those of the authors and do not necessarily represent those of their affiliated organizations, or those of the publisher, the editors and the reviewers. Any product that may be evaluated in this article, or claim that may be made by its manufacturer, is not guaranteed or endorsed by the publisher.
