# Supplementary material for: The Δ1-pyrroline-5-carboxylate synthetase family performs diverse physiological functions in stress responses in pear (Pyrus betulifolia)
Source: Front Plant Sci. 2022 Nov 24;13:1066765. doi: 10.3389/fpls.2022.1066765 (PMC9731112; doi:10.3389/fpls.2022.1066765)
Supplement: Supplementary file 1 [file DataSheet_1.docx]

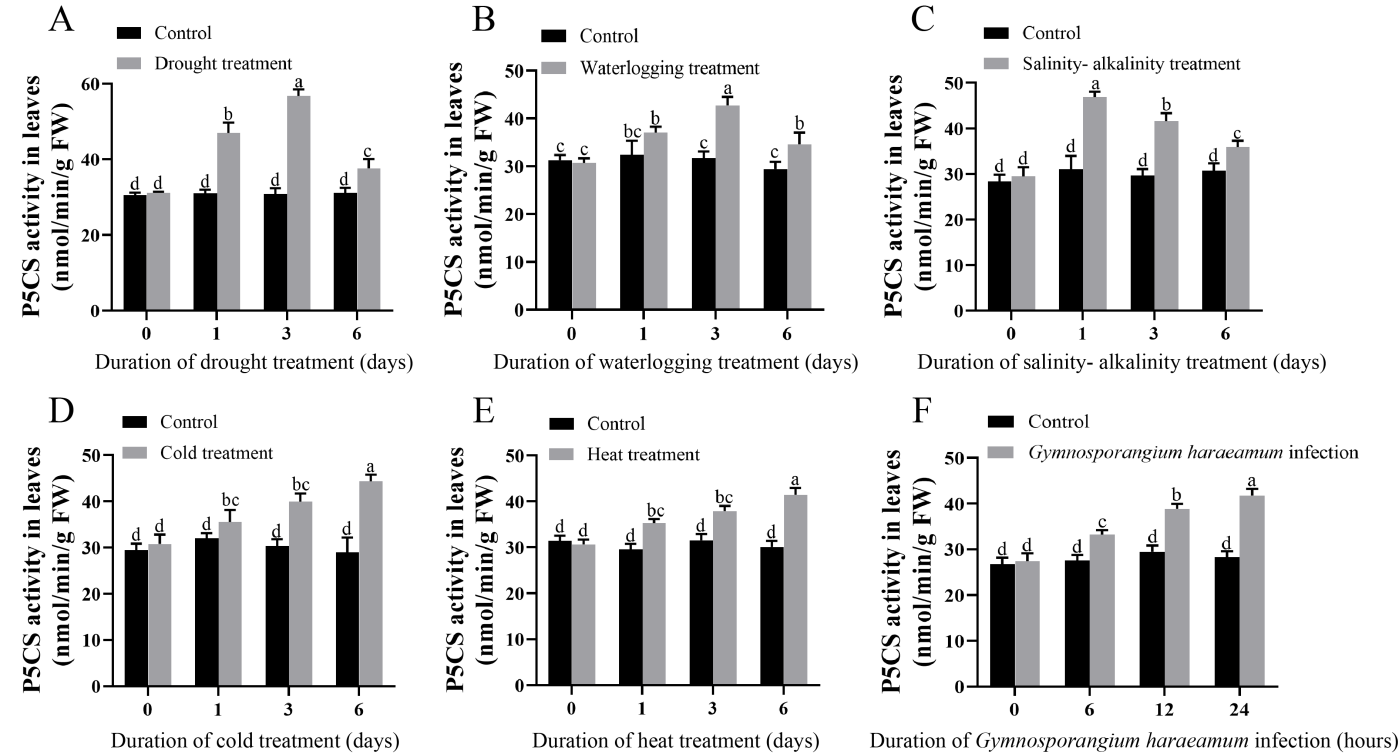


**SUPPLEMENTARY FIGURE S1 |** P5CS enzyme activity under (A) drought, (B) waterlogging, (C) salinity-alkalinity, (D) cold, (E) heat, and (F) *Gymnosporangium* *haraeanum* infection stresses. The data represent the mean ± SD of biological replicates. Different lowercase letters indicate significant differences according to Fisher’ s least significant difference (P< 0.05).
