# Supplementary material for: The Δ1-pyrroline-5-carboxylate synthetase family performs diverse physiological functions in stress responses in pear (Pyrus betulifolia)
Source: Front Plant Sci. 2022 Nov 24;13:1066765. doi: 10.3389/fpls.2022.1066765 (PMC9731112; doi:10.3389/fpls.2022.1066765)
Supplement: Supplementary file 2 [file Table_1.docx]

**List of qRT-PCR Primers**

| **Primers** | **Forward Sequences (5’-3’)** | **Reverse Sequences (5’-3’)** |
| --- | --- | --- |
| *PbP5CS1* | TTTAGTCAGTTGGACGTGTCGT | AAGAATAAGAAGATCAGCCTTTAGC |
| *PbP5CS2* | AAGACGGTAGAGCAGTGTAAGAATA | ACTTGGCTTTCAGGTTGTTTCC |
| *PbP5CS3* | TACAGTTCATTGGCTTGCTCAG | AACCCATCTTGTTGTTAGTAATCCTTCA |
| *PbP5CS4* | GTTTCAGAGGCACGACAAGCAG | TTCAGGACGGGACTCAAATACA |
| *PbP5CS5* | GTTGAGTGACAATGGCGGGTTT | GAATCCTGCTCGTACTTATCCC |
| *PbP5CS6* | TATGGACGAAAGCCTTGATGTT | CGGTCAGAGTTTGTCAGGGAGA |
| *PbP5CS7* | AGAAACTACAAGATTTAGGACCACC | GTTACCCGTTAGCTCATCAACA |
| *PbP5CS8* | TTACTGCCTATTTCTGCTCTTGC | CTCTTCCATCGCCATCCTGT |
| *PbP5CS9* | GAGAAATCGCTGTGGCTAAAGA | AATGCCCTCAAAGCATGGGTGG |
| *PbP5CS10* | TTCTGGACGCCTACCTGGACAA | AGGCCAGTGACTCGCCGAAACG |
| *PbP5CS11* | TATCCTCCTCCACCACCTCCTG | CCATTCCCACTCATTGTCACCC |
| *Actin* | TGGTGTCATGGTTGGTATGG | CAGGAGCAACACGAAGTTCA |
