# Supplementary material for: The Δ1-pyrroline-5-carboxylate synthetase family performs diverse physiological functions in stress responses in pear (Pyrus betulifolia)
Source: Front Plant Sci. 2022 Nov 24;13:1066765. doi: 10.3389/fpls.2022.1066765 (PMC9731112; doi:10.3389/fpls.2022.1066765)
Supplement: Supplementary file 3 [file Table_2.docx]

The motif sequences of PbP5CS proteins identified by MEME tool

| Name | Logo | Sequences | E-value | Sites | Width |
| --- | --- | --- | --- | --- | --- |
| Motif 1 | 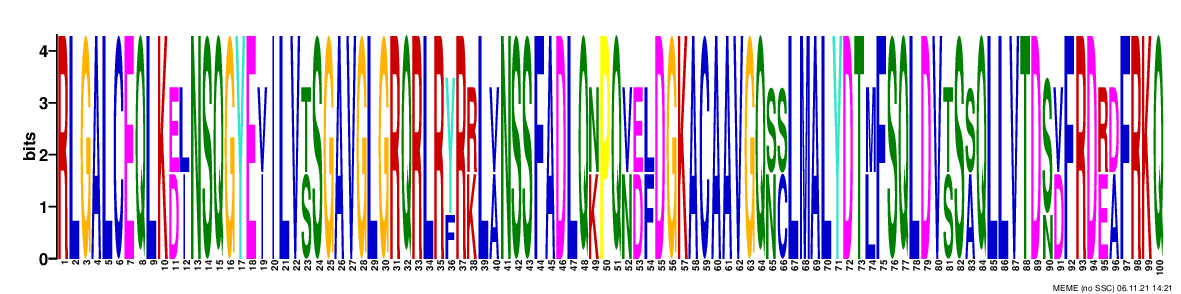 | RLGALCEQLKDJNSQGYEIILVSSGAVGLGRQRLRYRKLVNSSFADLQKPQNDFDGKACAAVGQNSLMALYDTLFSQLDVSSAQLLVTDSDFRDEDFRKQ | 5.0e-156 | 4 | 100 |
| Motif 2 | 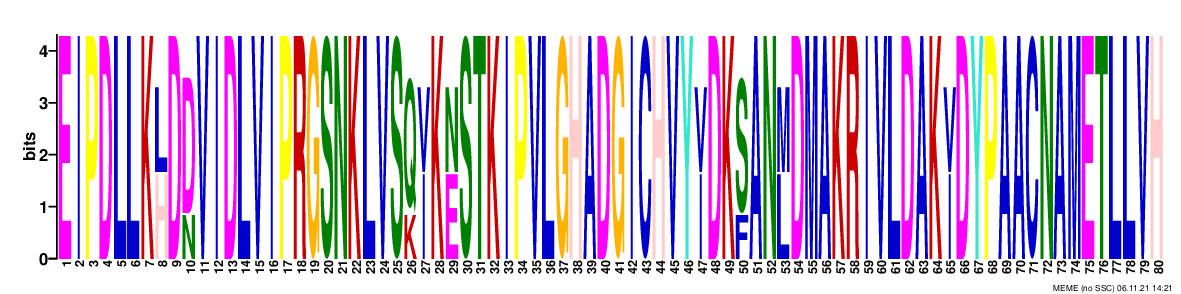 | EIPDLLKHDDVIDLVIPRGSNKLVSQIKESTKIPVLGHADGICHVYIDKSANLDMAKRIVLDAKIDYPAACNAMETLLVH | 1.3e-137 | 4 | 80 |
| Motif 3 | 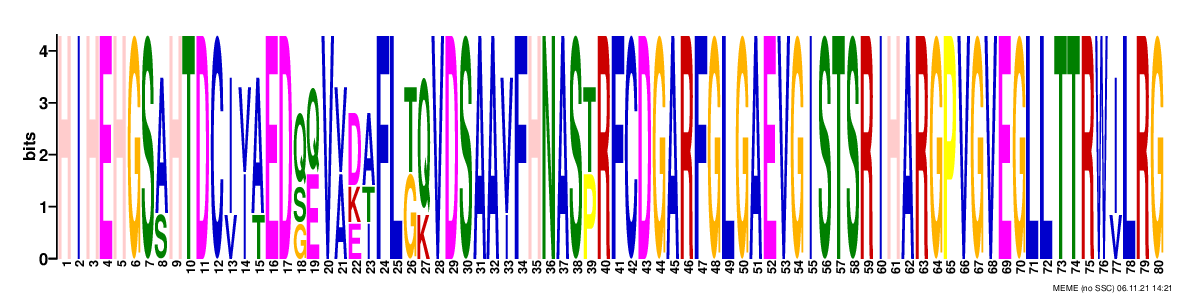 | HIHEHGSAHTDCIIAEDQZVVDAFLGQVDSAAVFHNASPRFCDGARFGLGAEVGISTSRIHARGPVGVEGLLTTRWILRG | 9.8e-130 | 4 | 80 |
| Motif 4 | 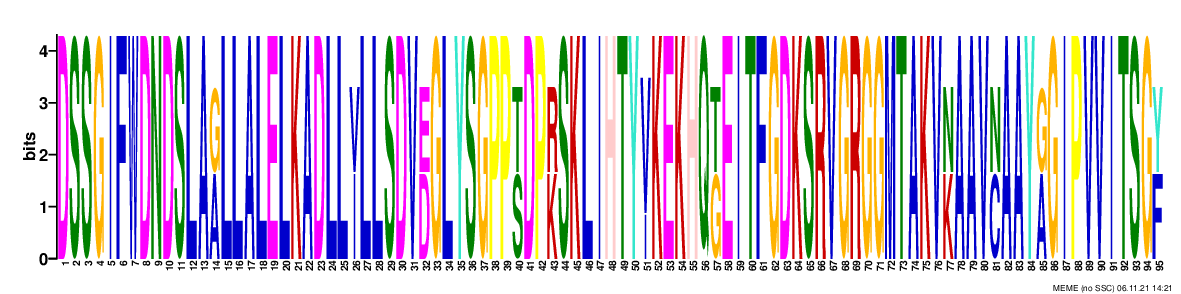 | DSSGIFWDNDSLAGLLALELKADLLILLSDVDGLYSGPPSDPKSKLIHTYVKEKHQGEITFGDKSRVGRGGMTAKVKAAVNAAYGGIPVVITSGY | 1.4e-150 | 4 | 95 |
| Motif 5 | 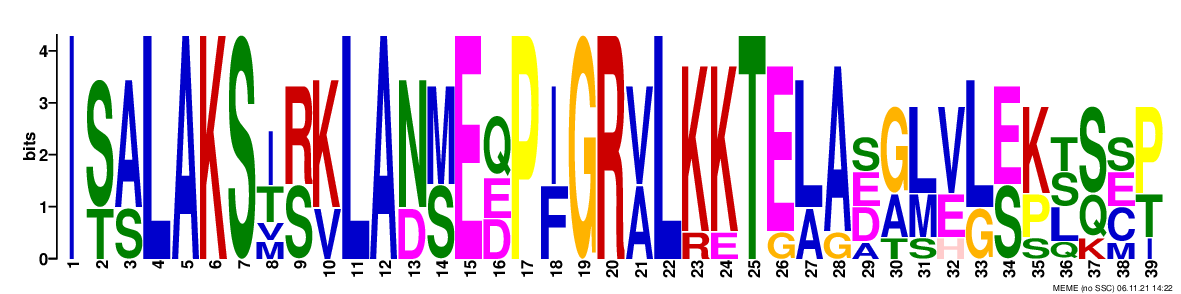 | ISALAKSIRKLANMEEPIGRVLKKTELAEGLVLEKTSSP | 7.3e-077 | 7 | 39 |
| Motif 6 | 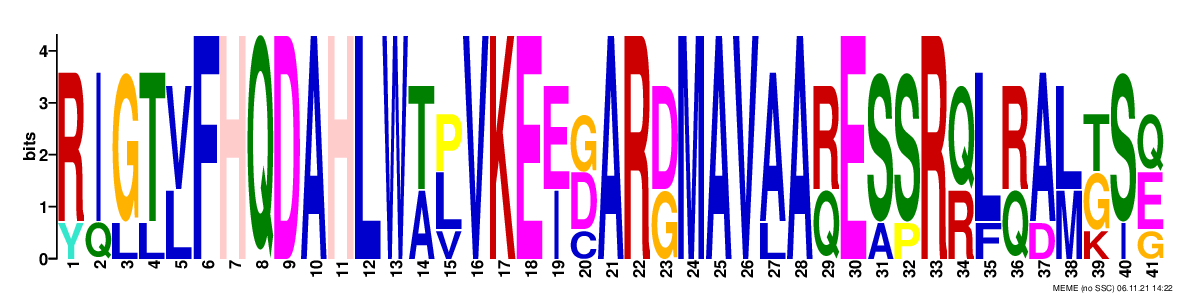 | RIGTVFHQDAHLWTLVKEEGARDMAVAARESSRQLRALGSZ | 1.4e-068 | 5 | 41 |
| Motif 7 | 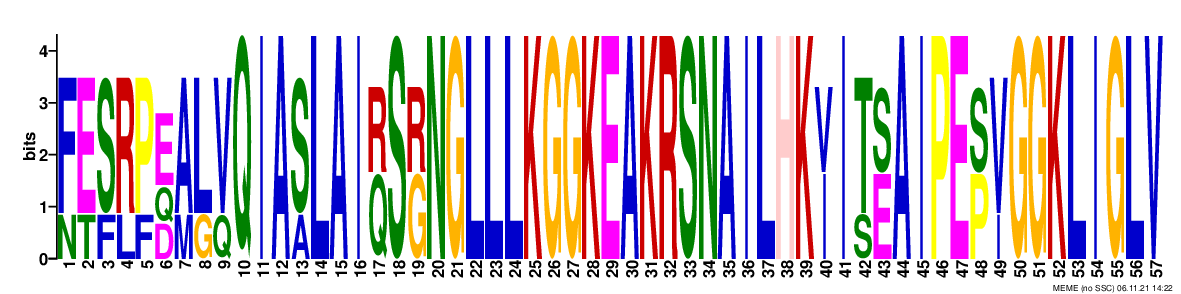 | FESRPEALVQIASLAIRSGNGLLLKGGKEAKRSNAILHKIITEAIPEPVGGKLIGLV | 9.8e-063 | 4 | 57 |
| Motif 8 | 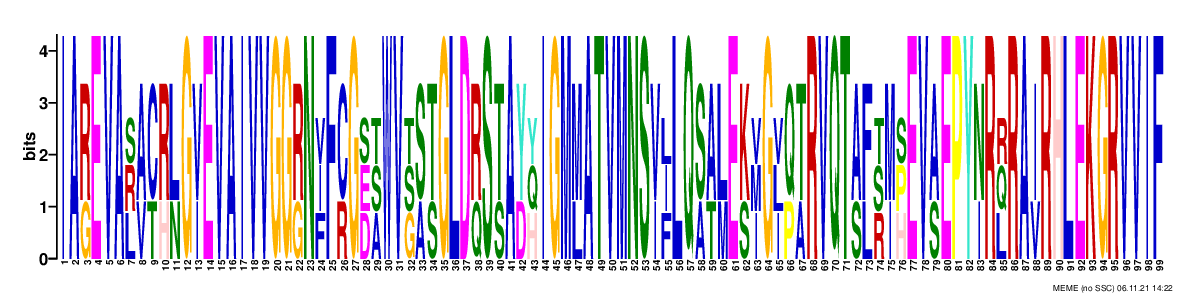 | IAREVARACRLGVEVAIVVGGRNIFCGDSWVSSTGLDRSTAYHIGMMATVMNSVLLQSALEKIGIQTRVQTAFSMPEVAEPYNRQRAIRHLEKGRVVIF | 7.5e-058 | 3 | 99 |
| Motif 9 | 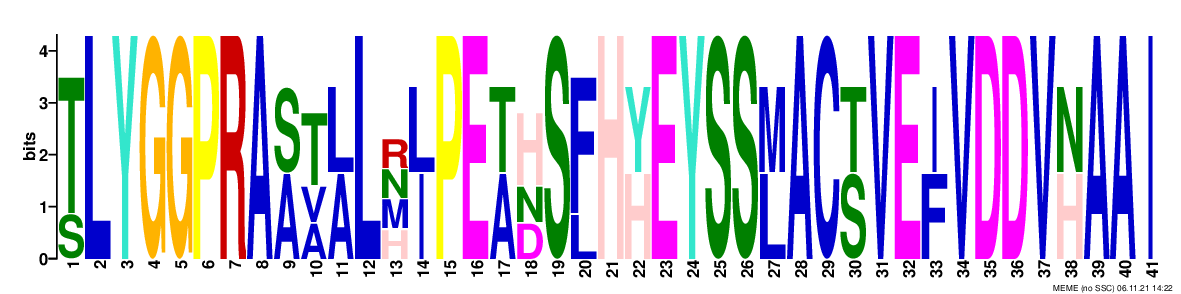 | TLYGGPRAATLLHJPEAHSFHHEYSSLACSVEFVDDVNAAI | 7.9e-050 | 4 | 41 |
| Motif 10 | 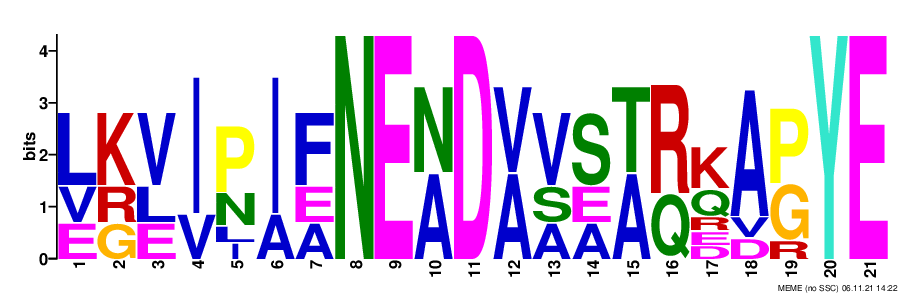 | LKVIPIFNEADVVSARKAPYE | 3.2e-040 | 8 | 21 |
